# Dormant carbohydrate reserves enhance pecan tree spring freeze tolerance: controlled environment observations

**DOI:** 10.3389/fpls.2024.1393305

**Published:** 2024-05-22

**Authors:** Amandeep Kaur, Lu Zhang, Niels O. Maness, Louise Ferguson, Charles J. Graham, Yanwei Sun, Srijana Panta, Niranjan Pokhrel, Ming Yang, Justin Q. Moss

**Affiliations:** ^1^ Department of Horticulture and Landscape Architecture, Oklahoma State University, Stillwater, OK, United States; ^2^ Department of Plant Sciences, University of California Davis, Davis, CA, United States; ^3^ Noble Research Institute, Ardmore, OK, United States; ^4^ Department of Plant Biology, Ecology, and Evolution, Oklahoma State University, Stillwater, OK, United States

**Keywords:** *Carya illinoinensis*, low temperature, scion/rootstock, sugars, starch

## Abstract

Pecan (*Carya illinoensis*), an economically important deciduous tree, bears commercially valuable nutritional nuts. Spring freezes in April can severely injure pecan buds, decreasing bloom, and fruit set. This study determined how low temperatures affect pecan buds/flowers at different growth stages in several pecan scion/rootstock combinations. This study focused on three pecan scion/rootstock combinations: Pawnee/Peruque (PP), Kanza/Giles (KG), and Maramec/Colby (MC), grown at the Cimarron Valley Research Station, Perkins, Oklahoma. Branches at three different growth stages, i.e., outer bud scale shed, one week after bud break, and early bloom stages were collected from PP, MC, and KG. Branches were held in a Conviron E8 freezing unit at 4 temperatures (-2, 0, 2, and 4°C) for 4 and 8 hours; A total of 8 treatments. One sample set was kept as an untreated control. After 2–3 weeks, branch samples from all the temperature treatments were observed and categorized into two groups. Group one with number of branches had healthy buds/formation of healthy leaves/flowers and group two with number of dead branches. The carbohydrate content reserved from dormant was analyzed using an Anthrone reagent. Visual observations and carbohydrate analyses revealed differences in damage and carbohydrate content among the scion/rootstock combinations, low-temperature treatments, and growth stages. The MC combination had minimum visual damage to leaves, buds, and flowers and significantly lower soluble sugars and starch in bark phloem as well as significantly lower soluble sugars in woody tissue xylem. The KG combination had maximum visual damage and significantly higher soluble sugars and starches in the bark, and soluble sugars in the woody tissues. These results indicate the MC combination is more tolerant to spring freeze damage at all three growth stages compared to the other two pecan scion/rootstock combinations. The results also demonstrate the MC combination is using more non-structural carbohydrates, soluble sugars and starches, suggesting this is a possible mechanism in its freeze tolerance.

## 1 Introduction

Pecan is a member of the *Juglandaceae* family, native to North America (Thompson and Conner, 2012). Pecan is a monoecious, heterodichogamous woody perennial and commercially important nut crop (Fayek et al., 2008; Cowell, 2015; Carroll and Smith, 2017; Andersen and Crocker, 2019). The different development stages in pecan include bud dormancy (November-February), bud dormancy break (March-April), flowering (end-April-May), fruit/nut set and development (May-October), nut harvesting (October-November), and leaf abscission and dormancy (November-February). Freeze damage in pecans can occur during different developmental stages. Spring freezes at flowering stage reduce potential crop (Malstrom et al., 1982). Fall freezes at shuck split inhibit shuck opening of mature nuts. Late winter freezes cause shoot dieback (Smith et al., 1993). The increasingly frequent late spring freezes are becoming more harmful to pecan production (Sparks and Payne, 1978; Kaur et al., 2020).

A fully dormant bud is maximally resistant to freezing temperatures (Farokhzad et al., 2018). In spring, buds swell and are more susceptible to freezing damage. Spring freeze damage is generally confined to terminal and primary buds which produce flowers and nuts. The damage to primary buds can be followed by growth of secondary and tertiary buds with lower crop potential than primary buds (Malstrom et al., 1982; Zhang et al., 2022). Malstrom et al. (1982), Reid (2014), and Reid (2020) all reported the bud, catkins, and pistillate flower damage after exposure to spring freeze (-5.5, -3.3, -1.1°C) in mid-April. Buds at different growth stages (outer bud scale intact, outer bud scale split, and outer bud scale shed) exposed to -6°C for 8 hours on 7 April 2009 had different bud mortality rate among cultivars (Smith and Cheary, 2010). For example, ‘Kanza’ (92%) had a higher bud survival rate than ‘Pawnee’ (45%). Bud survival rates declined at advanced bud growth stages in ‘Kanza’, 92% at outer bud scale intact, 78% at outer bud scale split, and 76% at outer bud scale shed stage.

Scion and rootstock combinations respond differentially to freezing temperatures depending upon their stage of growth (Grauke and Pratt, 1992). A ‘Sioux’ seedling rootstock was more severely damaged than ‘Burkett’ seedling at the inner scale split stage. Breaking bud dormancy late avoids spring freeze injury (Cade, 2001; Sakar et al., 2017). Rootstocks with early budding force early growth in scions. ‘Stuart’ seedlings break buds late, offering some spring freeze protection. ‘Elliott’ seedlings break buds late early, making them more susceptible to freeze damage than a ‘Moore’ rootstock (Grauke, 2019).

Determining critical temperatures for freeze damage is necessary to predict production losses (Matzneller et al., 2016). Controlled chamber experiments to determine the critical temperatures for damage in specific developmental stages and genotype in different *Prunus* species (Miranda et al., 2005; Matzneller et al., 2016; Szalay et al., 2018; Zhang et al., 2018), and to select frost resistant genotypes in Persian walnut and hazelnut (Baldwin, 2009; Panahi et al., 2021), have been conducted by multiple authors.

It is important to understand the effects of freezing on the tree’s metabolites, primarily the carbohydrates reserved from dormant. Carbohydrate metabolism has an important role in the floral induction and flowering in plants (Chen et al., 2018) and can enhance cold hardiness (Morin et al., 2007). The concentration of carbohydrates has been reported to influence hormone signaling pathways, such as gibberellic acid in Arabidopsis and abscisic acid in walnut (Juglans sigillata), thereby potentially regulating flower induction (Kanno et al., 2016; Lu et al., 2020; Kaur et al., 2021). High levels of carbohydrates before bud burst in spring are indeed associated with a higher yield in almond (Prunus dulcis), pistachio (Pistacia vera), and walnut (Zwieniecki et al., 2022) and a heightened occurrence of secondary bud burst in cases where primary buds were damaged by spring freeze in pecan (Zhang et al., 2023). Our previous study, focused on an April 21, 2021, 6-hour freeze with temperatures from -1.6 to -0.5°C, indicated damage to terminal leaves, buds, and catkins varied among cultivars and demonstrated an increase in bark soluble sugar levels after the freeze (Kaur et al., 2023). Understanding the role of carbohydrates in the low temperature responses of pecans at flowering could be a significant step towards a better freeze mitigation,

This study aims to identify the pecan scion/rootstock combination with the highest freeze tolerance across three growth stages and explore the impact of stored non-structural carbohydrates in the shoots on the spring freeze tolerance of buds/flowers.

## 2 Materials and methods

Three pecan scion/rootstock combinations used in the experiments were Pawnee/Peruque (PP), Kanza/Giles (KG), and Maramec/Colby (MC), from 27-year-old trees grown at the Cimarron Valley Research Station (97°02’13” W 35°58’55” N), in Perkins, Oklahoma. Branch samples (30 ± 2 cm in length) for treatment were selected from the middle part of the canopy of approximately same height at three growth stages: stage I (outer bud scale shed stage), stage II (one week after outer bud scale shed stage), and stage III (early bloom stage). Branches were placed immediately into water to prevent desiccation, transferred to the Oklahoma State University campus in Stillwater and placed into a Conviron, CMP3244 growth chamber (Conviron, Controlled Environmental Research Laboratories, OSU, Stillwater) to await low temperature treatment. Temperature, humidity, and light conditions approximated spring conditions at the site (**Supplementary Table 1**). Branches collected at each growth stage were treated with cold treatments one day after sampling. A set of nine branches from each cultivar PP, KG, and MC were treated with 4 different temperatures (-2, 0, 2, and 4°C) in the dark using a Conviron E8 Freezing Unit at two different durations (4 and 8 hours). During low temperature treatment, the temperature of freezing unit was lowered gradually from 12°C (night temperature of the growth chamber) to treatment temperature by decreasing 3°C per hour. One set of nine branches remained as a control in the growth chamber (with conditions provided in **Supplementary Table 1**) without low temperature treatment. Water was changed twice per week. Cold-treated and control branches were kept in the growth chamber for an additional 2–3 weeks waiting for regrowth after the temperature treatments (to observe how they respond and grow) and observed visually.

### 2.1 Visual observations

**Figure 1:**
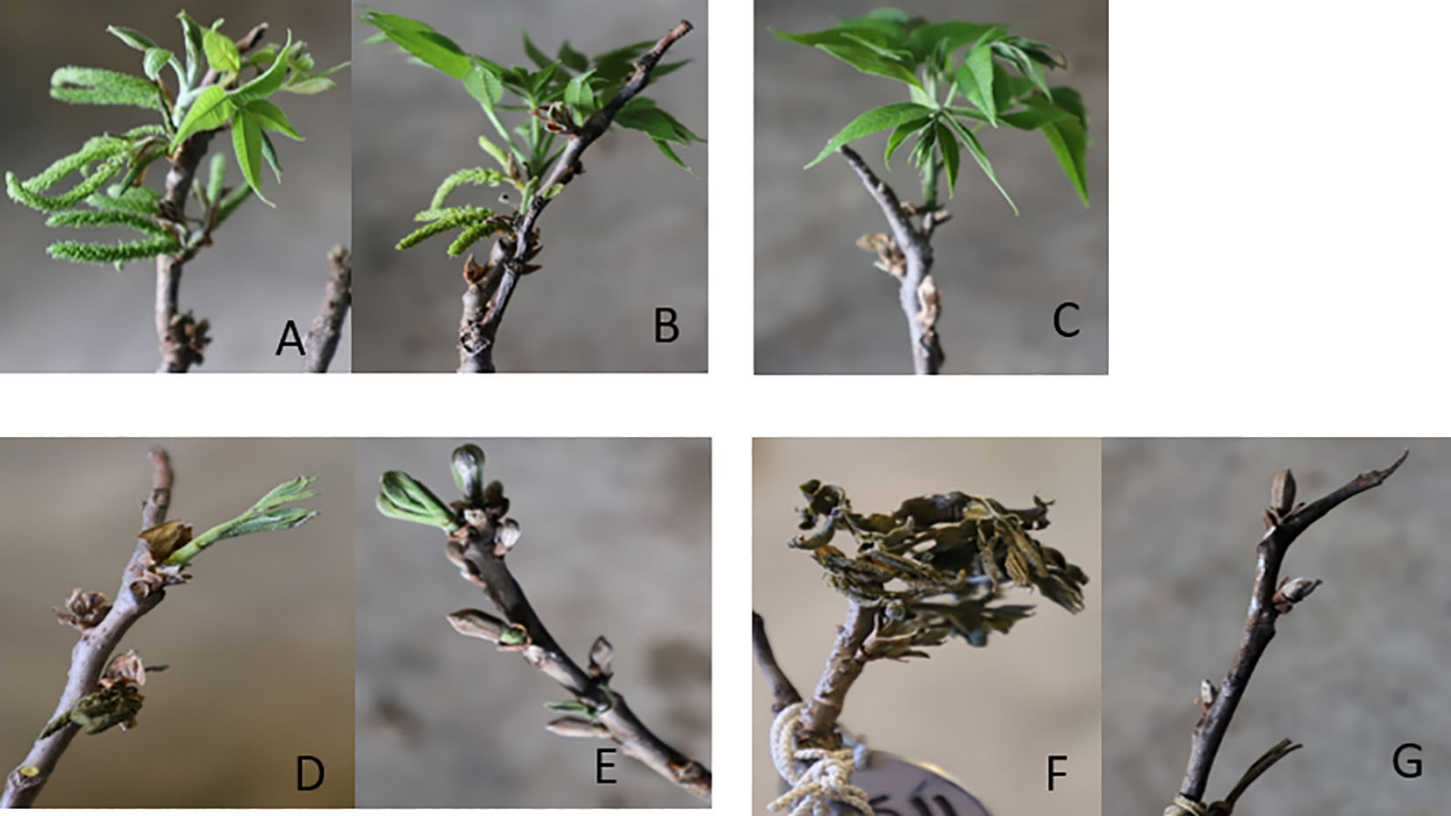
To visually observe and compare the damage after the temperature treatments, branches were divided into two categories: number of branches with live male catkins **(A, B)** /green healthy leaves **(C)** /terminal green or live bud **(D, E)** and number of branches dead **(F, G)**.

For the visual observations, nine branches per each scion/rootstock combination (PP, MC, and KG) for each temperature treatment/duration combination (i.e., -2, 0, 2, and 4°C for 4 and 8 hours) at three stages (stage I, stage II, and stage III) were used. To visually observe and compare the damage after the temperature treatments, branches were divided into two categories: number of branches with live male catkins/terminal bud/green healthy leaves (**Figures 1A–E**) and number of branches dead (**Figures 1F, G**).

### 2.2 Soluble sugar and starch analysis of bark and wood

Branches of three pecan scion/rootstock combinations (PP, MC, and KG) kept in the growth chamber after different low-temperature treatment (from 8 hours duration) were used for soluble sugar and starch analysis of bark (containing phloem vascular tissue) and wood (containing xylem vascular tissue) tissue. The nine branches per scion-rootstock combinations for each temperature treatment were used. The top section (4–5cm in length from the terminal bud) from each of the branches was used. The wood and bark of each section were separated manually, chopped using pruning shears, and dried in the oven (Isotemp oven Model 655F, Fisher Scientific, USA) for 2–3 days at 75°C. After drying, the samples were kept at room temperature until further processing. The fine powder (25–27 mg) bark and wood tissues were further processed for soluble sugars and starch analysis using colorimetric Anthrone regent method (Kaur et al., 2023).

### 2.3 Statistical analysis

For soluble sugars and starch, total carbohydrate content was determined based on glucose equivalents and were expressed as mg g^-1^ DW (dry weight). The data were analyzed using PROC GLM in Statistical Analysis System (Version 9.4; SAS Institute Inc., Cary, NC). Three-way analysis of variance (ANOVA) was performed to determine the effects of low temperature on soluble sugar and starch concentration in different scion/rootstock combinations at three stages, and the treatment differences were analyzed using the LSMEANS with LINES statement at α = 0.05.

## 3 Results

### 3.1 Visual observations: low-temperature damage among scion/rootstock depends upon growth stage at time of treatment

**Figure 2:**
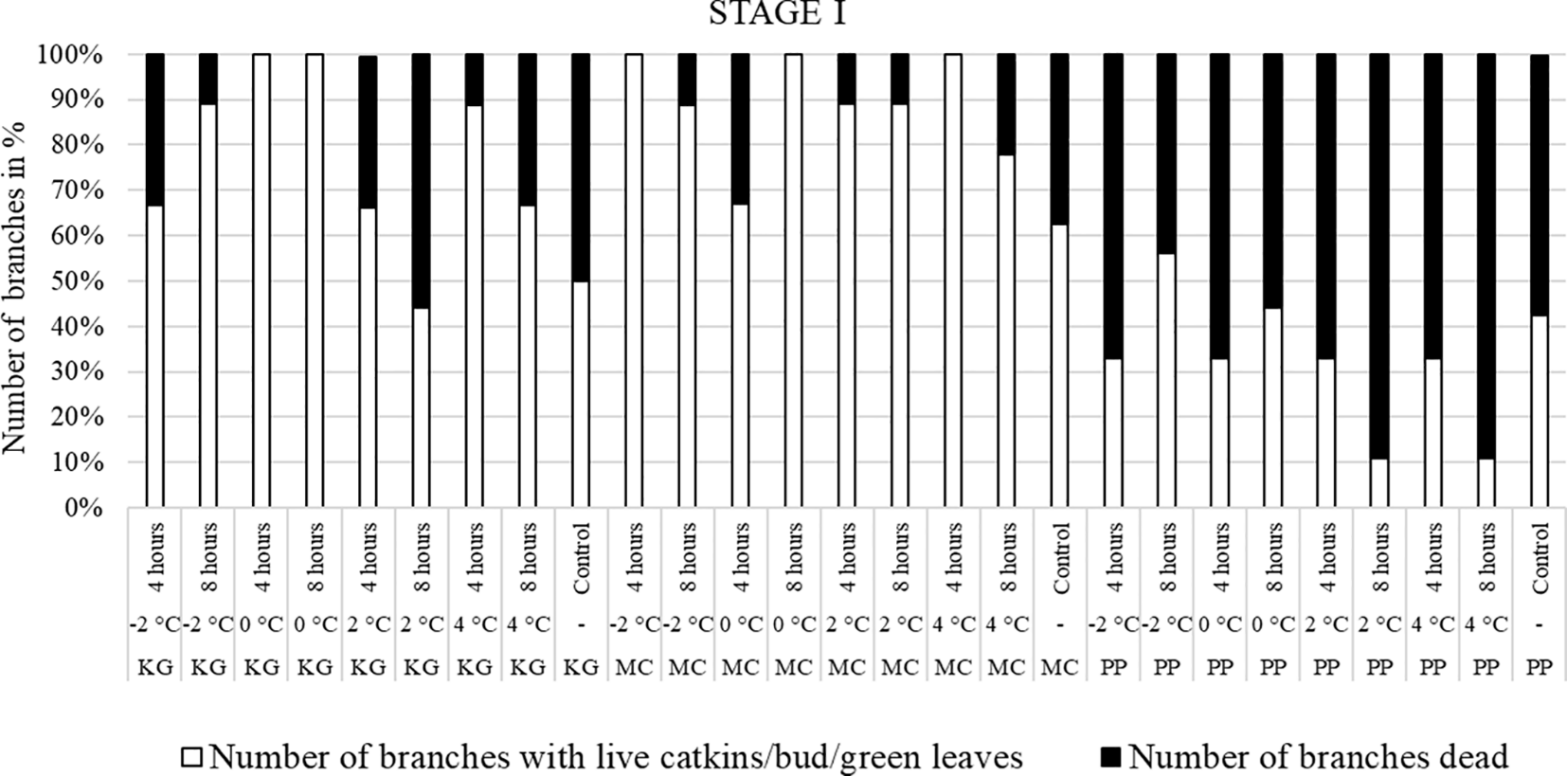
Visual observation of stage I (outer bud scale shed stage): Branches after treating with four different low-temperatures for 4 or 8 hours and controls of three pecan scion/rootstock combinations KG (Kanza/Giles), MC (Maramec/Colby), and PP (Pawnee/Peruque).

**Figure 3:**
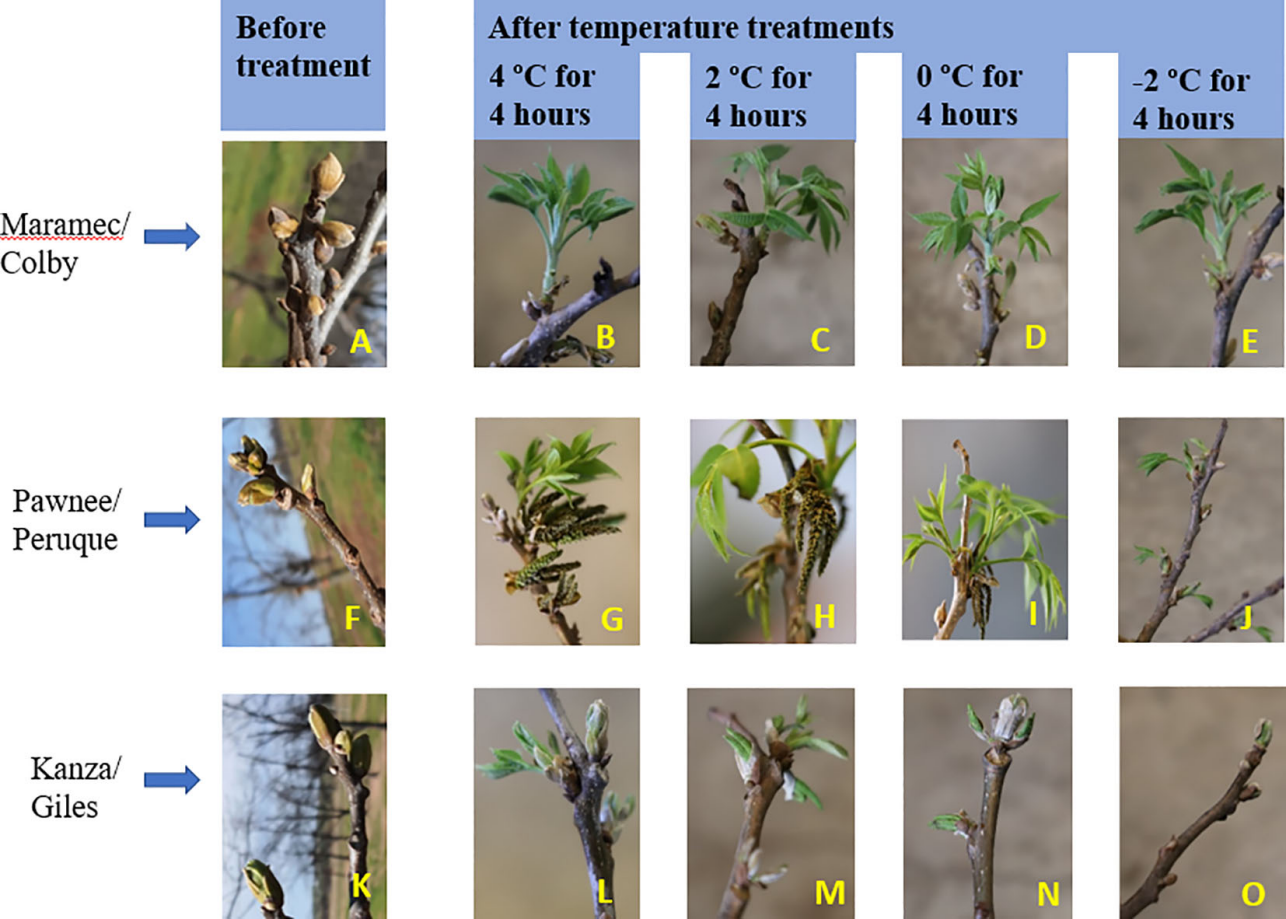
Visual observation of stage I (outer bud scale shed stage): Branches before treatment and after treated with four different low-temperatures from three pecan scion/rootstock combinations Maramec/Colby **(A-E)**, Pawnee/Peruque **(F-J)**, and Kanza/Giles **(K-O)**.

**Figure 4:**
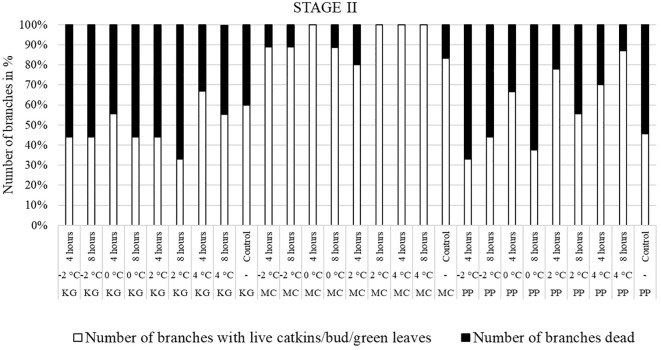
Visual observation of stage II (one week after bud break): Branches after treating with four different low-temperatures for 4 or 8 hours and controls of three pecan scion/rootstock combinations KG (Kanza/Giles), MC (Maramec/Colby), and PP (Pawnee/Peruque).

**Figure 5:**
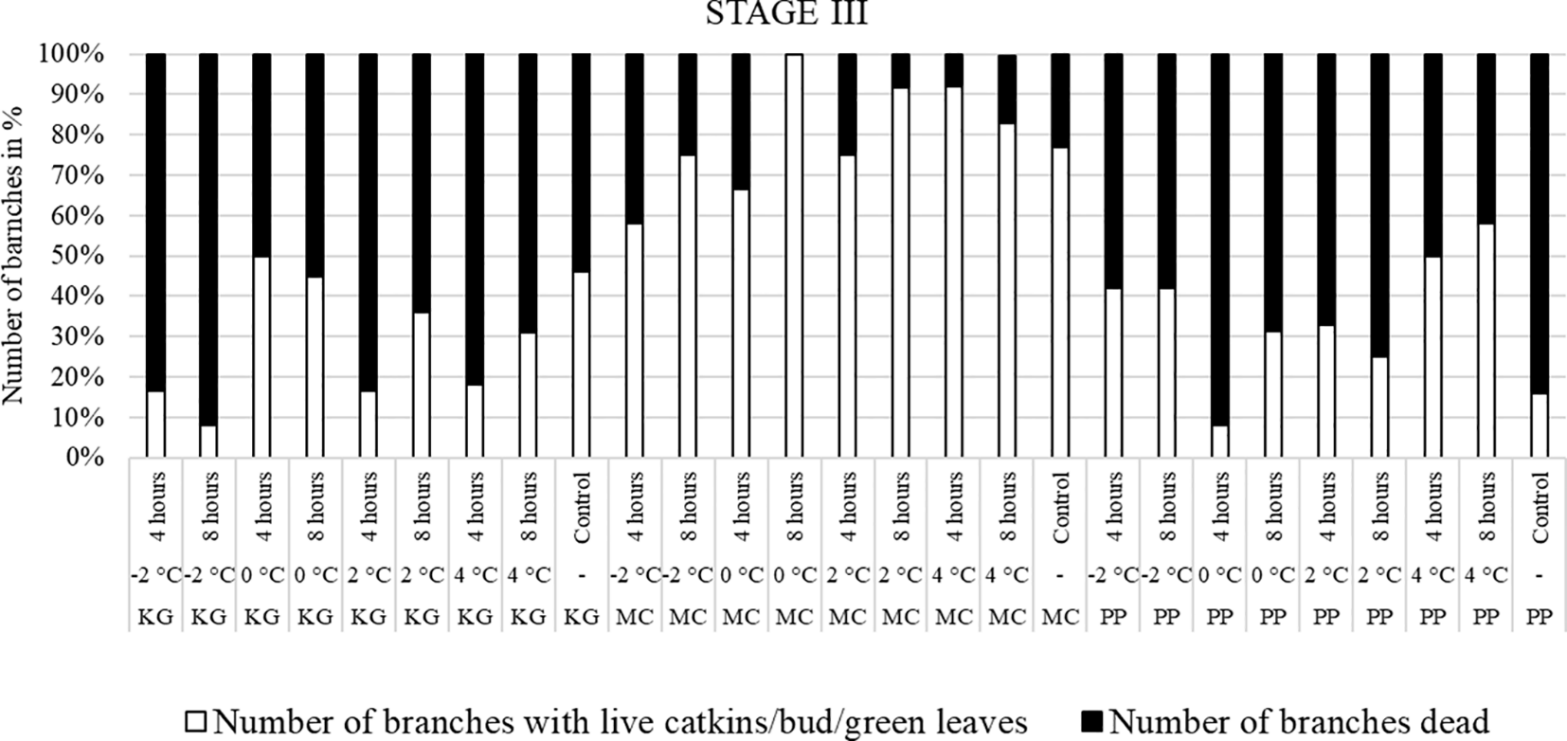
Visual observation of stage III (early bloom): Branches after treating with four different low-temperatures for 4 or 8 hours and controls of three pecan scion/rootstock combinations KG (Kanza/Giles), MC (Maramec/Colby), and PP (Pawnee/Peruque).

Branches collected from three pecan scion/rootstock combinations at stage I (outer bud scale shed stage) and stage II (a week after outer bud scale shed stage) were observed three weeks after the low temperature treatments (**Figures 2**–**5**). Post cold treatment most Maramec/Colby (MC) branches produced large leaves and catkins (**Figures 2**, **3B-E**). The Pawnee/Peruque (PP), branches also produced leaves and catkins, but most branches were dead on the day of observations (**Figures 2**, **3G-J**). Kanza/Giles (KG) at all the temperature treatments and controls, had bud break and catkins but catkins were very small (**Figures 3L-N**). KG branches treated for 4 hours at temperatures (4, 2, and 0°C) had better leaf or catkin growth than branches treated for 8 hours. Overall, when branches were treated at stage I, most KG branches had only terminal live buds or small catkins, MC branches had large green leaves, and PP had dead branches.

In KG, most branches were dead after all low-temperature treatments (except 4°C for 4 hours) at stage II (**Figure 4**). Only a few branches of KG produced leaves. Most MC branches produced leaves after treatment. In PP, most branches treated at -2°C for 4 and 8 hours were dead. The percentage of dead branches declined with higher temperature treatments. In 0°C and 2°C treated, PP branches treated for 4 hours had most of branches produced male catkins and green leaves compared to 8 hours treatment. PP branches treated with 4°C for 4 and 8 hours had more leaves and catkins and less percentage of dead branches. Overall, most KG branches treated at stage II were dead or with terminal green buds only whereas MC had green healthy leaves. In PP, many branches were dead when treated at -2°C, and the percentage of dead branches declined with higher temperature treatments. In PP, most of branches treated at 4°C had live male catkins and leaves.

The branches treated with low temperatures at stage III (early bloom stage) were visually observed after two weeks of treatment (**Figure 5**). In KG and PP, most of branches had dead leaves and catkins after low-temperature treatments. Whereas most of the MC branches had green leaves or terminal green buds after temperature treatments, but they easily dislodged and fell when touched.

Overall, the branches collected at stage III showed the most damage, followed by stage II, while the branches treated at stage I had the least damage.

### 3.2 Soluble sugar and starch in pecan bark and wood tissue

The three-way scion/rootstock × treatments × stage interaction was significant in bark soluble sugar, bark starch, woody tissue soluble sugar, and woody tissue starch content (**Table 1**).

**Table 1:** Summary ANOVA table for bark soluble sugar (mg g-1 DW), bark starch (mg g-1 DW), wood soluble sugar (mg g-1 DW), and wood starch (mg g-1 DW) content in pecans.

Source	Bark Soluble Sugar	Bark Starch	Wood Soluble Sugar	Wood Starch
Scion/rootstock (SR)	***	***	***	*
Treatment (T)	***	***	***	***
SR×T	***	***	***	**
Stage (S)	***	***	***	***
SR×S	***	NS	***	***
T×S	***	***	***	***
SR×T×S	***	***	***	***

*** significant at 0.001, ** significant at 0.01, * significant at 0.05, NS, nonsignificant.

#### 3.2.1 Soluble sugar content in pecan bark

**Figure 6:**
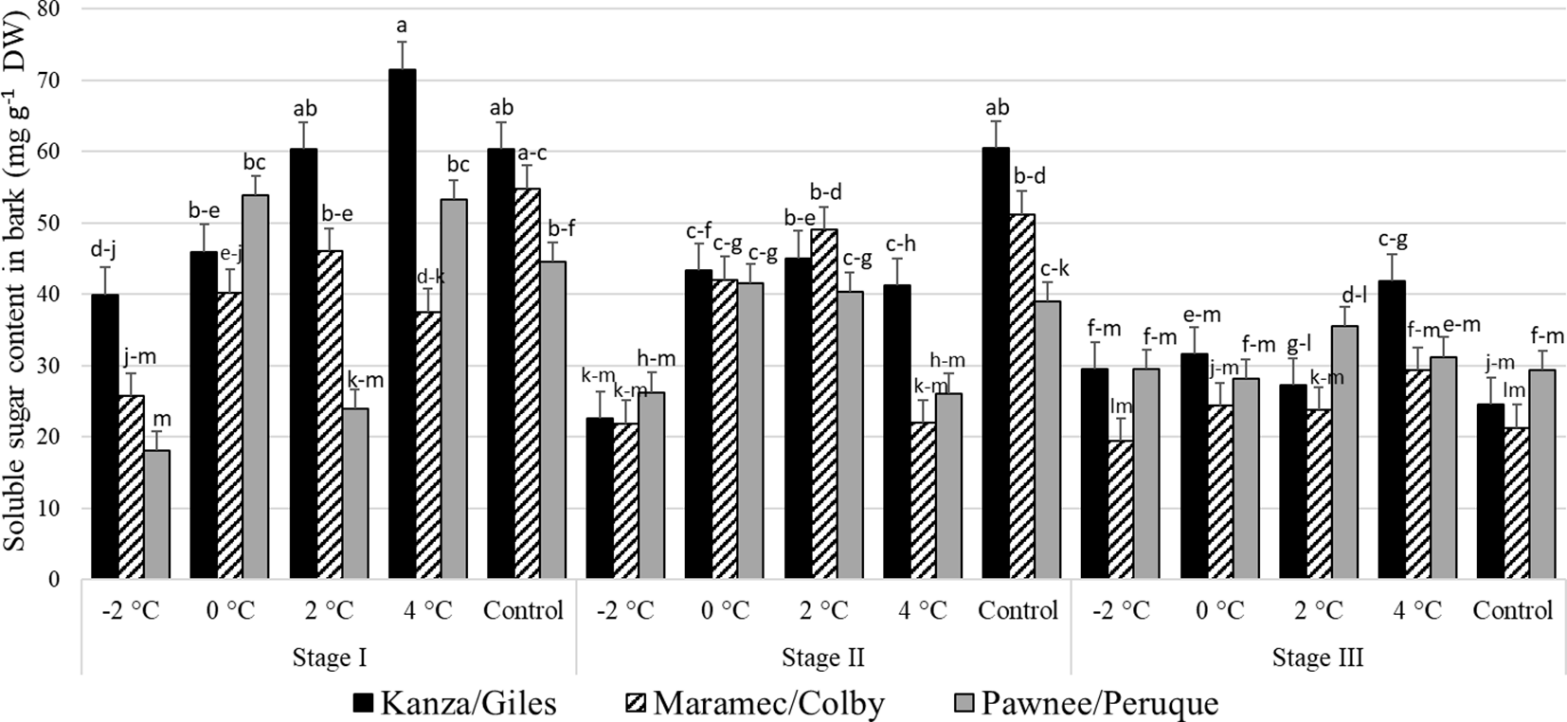
Soluble sugar concentration in bark for four different temperature treatments (-2, 0, 2, and 4°C for 8 hours) and controls of three pecan scion/rootstock combinations Kanza/Giles, Maramec/Colby, and Pawnee/Peruque at three growth stages (stage I: outer bud scale shed stage, stage II: one week after outer bud scale shed stage, and stage III: early bloom stage). Means with the same letter are not significantly different at P<0.05. Bars above each treatment represent the standard error for the mean of nine replications.

Stage I; outer bud scale shed stage: In KG, the bark sugar content in branches treated with -2°C was significantly lower than branches treated with 2°C, 4°C, and controls (**Figure 6**). In MC, -2°C treated branches had significantly lower bark sugar concentration compared to those treated with 2°C and controls. Similarly, in PP and the control, 4°C, and 0°C branches had significantly higher sugar levels compared to -2°C treated branches. There were no significant differences in sugar content among the three untreated (controls) pecan scion/rootstock combination. However, there were significant differences among the scion/rootstock combinations treated with low-temperature treatments. For example, 4°C treated MC branches had significantly lower sugar in the bark compared to KG and PP.

Stage II; a week after outer bud scale shed stage: In KG and MC, controls, the 2°C, and 0°C treatments produced significantly higher bark sugar levels than those treated at -2°C. There were no significant differences among the bark sugar concentrations in PP branches from all temperature treatments and controls. Controls and 4°C treated branches showed significant differences among scion/rootstock combinations. For example, KG branches had significantly higher sugar than MC branches treated at 4°C.

Stage III; early bloom stage: In the KG, MC, and PP combination there were no significant differences in the bark soluble sugar content between treatments and controls as well as among scion/rootstock combinations with one significant difference. At 4°C the PP combination had significantly higher bark soluble sugar content than MC at -2°C. Overall, at stage III, soluble sugar content was significantly lower in MC compared to KG and PP.

There were significant differences among stage I, stage II, and stage III bark sugar concentrations in PP and KG. Stage I samples from KG treated with 4°C had significantly more soluble sugars compared to stage II and stage III samples.

#### 3.2.2 Starch content in pecan bark

**Figure 7:**
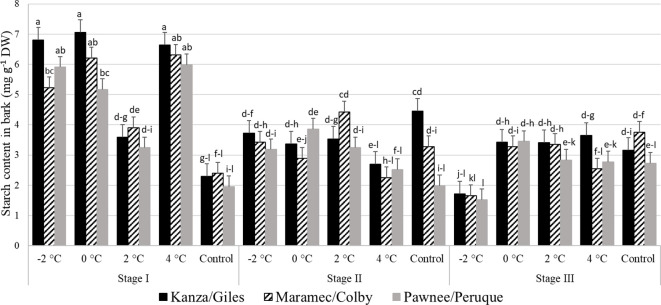
Starch concentration in bark for four different temperature treatments (-2, 0, 2, and 4°C for 8 hours) and controls of three pecan scion/rootstock combinations Kanza/Giles, Maramec/Colby, and Pawnee/Peruque at three growth stages (stage I: outer bud scale shed stage, stage II: one week after outer bud scale shed stage, and stage III: early bloom stage).Means with the same letter are not significantly different at P<0.05. Bars above each treatment represent the standard error for the mean of nine replications.

Stage I: In all three scion/rootstock combinations, the bark starch concentration in branches treated with 4°C, 0°C, and -2°C was significantly higher than branches treated with 2°C and controls (**Figure 7**). There were no significant differences in starch content among untreated control samples of the three-pecan scion/rootstock combinations. However, there were significant differences among the scion/rootstock combinations treated with low temperatures of 0°C and -2°C. For example, the -2°C treated MC branch had significantly lower bark starch content compared to KG branches.

Stage II: In KG, control branches had significantly higher starch levels in the bark than those treated with 4°C temperature. There was significant variation in MC branches treated with different low temperatures. A 2°C treated branches had higher starch content in the bark compared to 0°C and 4°C treated branches. For PP, only 2°C treated and control branches differed significantly in starch levels. Additionally, control branches had significant differences among the scion/rootstock combinations. For example, KG branches had significantly higher bark starch levels compared to PP branches.

Stage III: The KG -2°C treated branches had significantly lower starch content than all other treatments and controls. In MC, the bark starch content was significantly lower in -2°C treated branches compared to 0°C, 2°C, and control branches. Similarly in PP, the bark starch content in -2°C treated samples was also significantly lower than 0°C, 2°C, and 4°C samples.

There were significant differences between stage I, stage II, and stage II bark starch concentration in all the scion/rootstock combinations branches treated with -2°C and 4°C. Whereas in the controls, stage II KG samples had significantly higher starch content than stage I KG controls.

#### 3.2.3 Soluble sugar content in pecan wood

**Figure 8:**
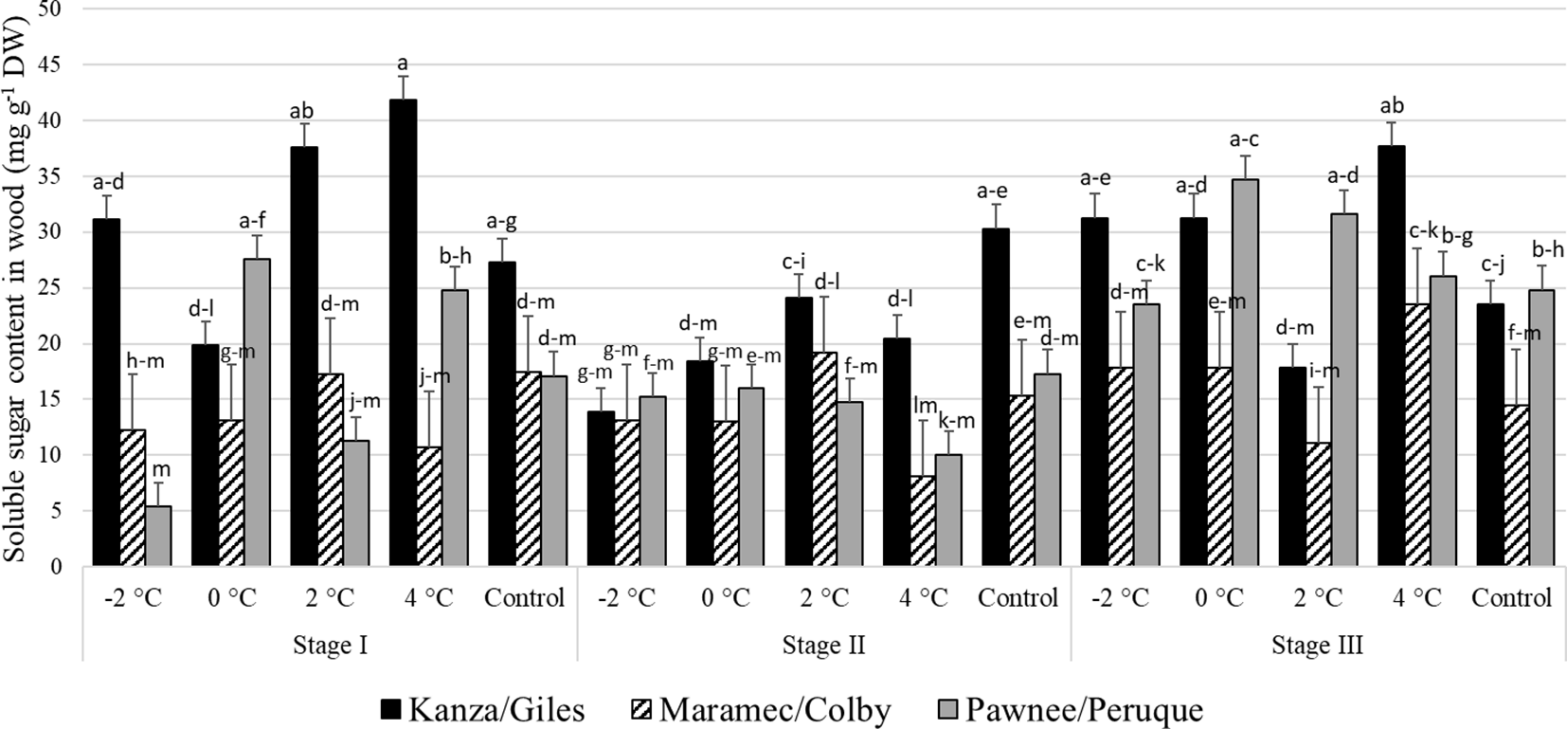
Soluble sugar concentration in woody tissue for four different temperature treatments (-2, 0, 2, and 4°C for 8 hours) and controls of three pecan scion/rootstock combinations Kanza/Giles, Maramec/Colby, and Pawnee/Peruque at three growth stages (stage I: outer bud scale shed stage, stage II: one week after outer bud scale shed stage, and stage III: early bloom stage).Means with the same letter are not significantly different at P<0.05. Bars above each treatment represent the standard error for the mean of nine replications.

Stage I: In KG, the woody tissue soluble sugar content in branches treated with 4°C and 2°C was significantly higher than branches treated with 0°C (**Figure 8**). There were no significant differences in soluble sugar contents among control samples of the three scion/rootstock combinations. However, there were significant differences among the scion/rootstock combinations treated with low-temperature treatments.

Stage II: In KG, control branches had a significantly higher woody tissue soluble sugar levels than those treated at all low temperatures. Further, there were no significant variations in the woody tissue sugar levels among PP, KG, and MC treated at low-temperature and control samples, except for the 4°C treatment. At 4°C, KG had significantly higher soluble sugar content than PP samples.

Stage III: At this stage also, only KG had significant differences in woody tissue soluble sugar content among the different low temperature treated and control samples. There were significant differences among the scion/rootstock combinations treated with 0°C and 2°C.

There were significant differences between stage I and stage II woody tissue soluble sugar concentration in KG and PP. Stage I branches from KG treated with 4°C had significantly more woody tissue sugar content compared to stage II branches. MC branches from stage I and stage II had no significant variation in woody tissue soluble sugar content between temperature treatments and controls. PP and MC exhibited no significant differences at all three stages in soluble sugar concentration of woody tissue from all temperature treatments and controls.

#### 3.2.4 Starch content in pecan wood

**Figure 9:**
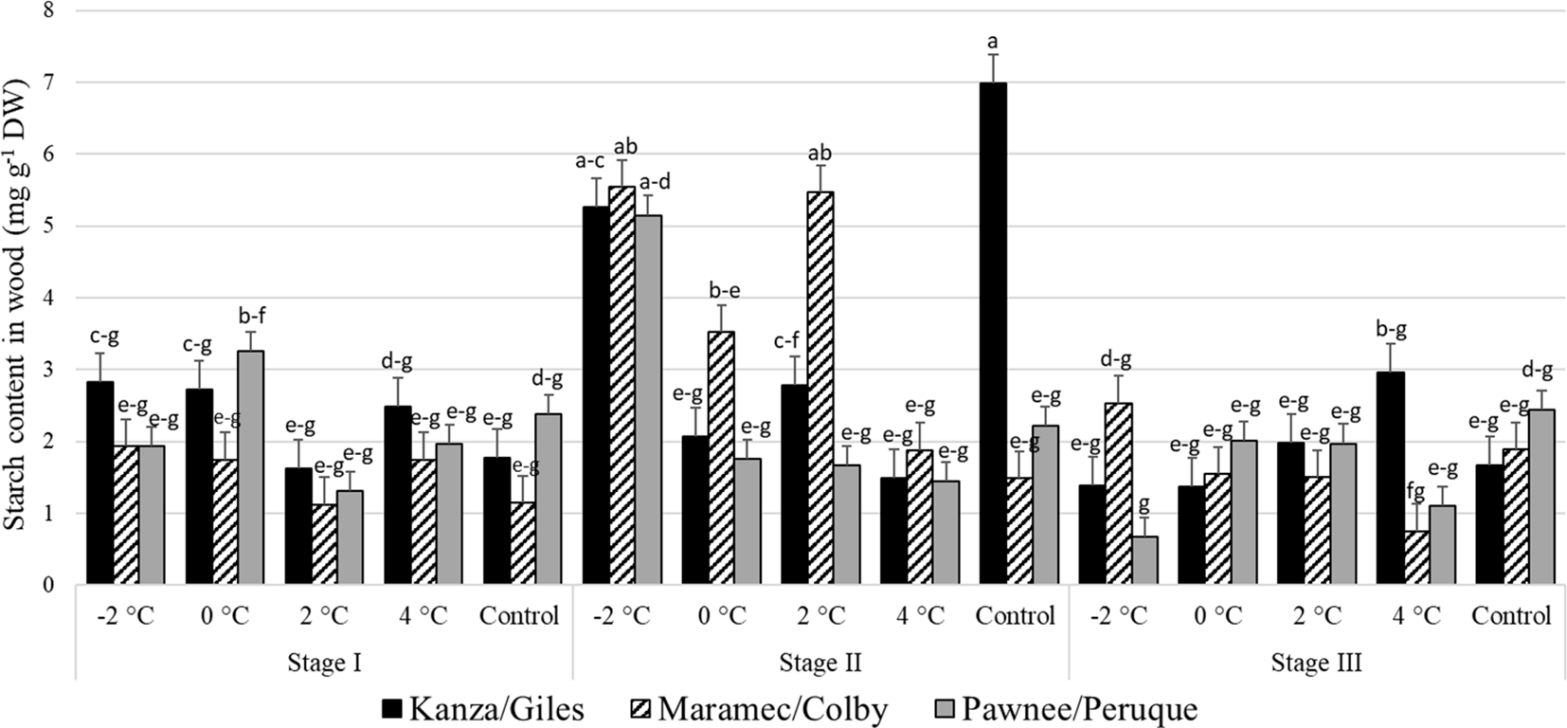
Starch concentration in woody tissue for four different temperature treatments (-2, 0, 2, and 4°C for 8 hours) and controls of three pecan scion/rootstock combinations Kanza/Giles, Maramec/Colby, and Pawnee/Peruque at three growth stages (stage I: outer bud scale shed stage, stage II: one week after outer bud scale shed stage, and stage III: early bloom stage).Means with the same letter are not significantly different at P<0.05. Bars above each treatment represent the standard error for the mean of nine replications.

Stage I and stage III: In all three scion/rootstock combinations, the woody tissue starch concentration in branches treated with 4, 2, 0, -2°C temperatures and controls were not significantly different (**Figure 9**). There were no significant differences in starch content in control branches or the low temperature treated branches among the scion/rootstock combinations.

Stage II: In KG, the control branches had significantly higher woody tissue starch levels than those treated at 4°C and 0°C. There was significant variation in MC branches treated at low temperatures; the controls and 4°C treated set woody tissue starch content was significantly lower than those treated at 2°C and -2°C. In case of PP, only the -2°C treated branches significantly differed from others in the starch levels. Additionally, the controls and 2°C treated branches had significant differences among the scion/rootstock combinations in woody tissue starch content. For example, control KG branches had significantly higher woody tissue starch levels compared to PP and MC branches.

There were significant differences between stage I and stage II woody tissue starch concentrations in the branches treated with -2°C, 2°C, and controls. For example, -2°C treated branches from stage I and stage III in PP and MC had lower starch content than those treated at stage II. Similarly, at stage II, KG control branches had significantly higher starch content than stage I KG controls.

## 4 Discussion

### 4.1 Visual observations

Low spring temperatures damage the soft fresh tissues of developing buds, new leaves, and reproductive organs (Hosseinpour et al., 2018). This study demonstrated branches at stage III early bloom, were the most severely damaged by all low temperatures compared to stage II and stage I samples. Freeze/frost injury is highly dependent on the phenological growth stage of the bud/flower (Aygun and San, 2005; Hosseinpour et al., 2018). In pecans, freeze induced minimal harm to dormant or enlarged buds, but caused significant damage to buds during the leaf expansion (Grauke and Pratt, 1992; Sparks, 1992; Farokhzad et al., 2018). Stage III had expanding leaves and flowers, which made them more susceptible to low temperatures compared to stage I and stage II.

Differences in damage/injury were observed among the scion/rootstock combinations after low temperature exposure. Overall, MC branches had the lowest mortality rates across all three growth stages compared to the other two scion/rootstock combinations. It might be because Maramec was the variety originally selected from the experimental orchards at OSU Cimarron Valley Research Station in Perkins, OK, while the other two varieties, Pawnee and Kanza, were released in the southern state of Texas (Smith, 2002; McCraw et al., 2014). Although all three cultivars are cold hardy and adaptable to the northern area, Maramec exhibits better tolerance to spring freezes. Low temperature tolerance varies among buds/flowers of different cultivars and species (Hosseinpour et al., 2018). For example, the severity of springtime low temperature damage varies among genotypes/cultivars in almonds, apples, and sour cherry (Aygun and San, 2005; Szpadzik et al., 2009; Imani et al., 2012).

There was also a difference in damage between the 4-hour and 8-hour duration temperature treatments. The longer 8-hour low temperature treatments caused more mortality than the 4-hour treatments. The extent of damage has been reported to be was directly related to intensity and duration low temperatures (Larsen, 2010).

The selection of superior cultivars is generally based on climatic adaptation, high production, and good kernel and nut quality. Spring freeze damage is an important limiting factor in tree nut production of pecans, walnut (Khadivi et al., 2019), pistachio (Nobari et al., 2012), almond (Imani et al., 2012). Evaluation and understanding of spring freeze damage among cultivars and rootstocks is important for the selection of trees with a broader climatic adaptation (Grauke and Pratt, 1992; Imani and Mahamadkhani, 2011; Khadivi et al., 2019).

### 4.2 Carbohydrate analyses

The soluble sugar contents in the three pecan scion/rootstock combinations were different after low-temperature treatments. However, the pattern of change was similar among the combinations. In this study, we observed significant decreases in the bark soluble sugar levels in stages I and stage II of branches treated with low temperatures (**Figure 6**). The MC bark had significantly reduced soluble sugars after a 4°C temperature treatment at stage I and stage II. It also had better growth than the other two scion/rootstock combinations (PP and KG) after a 4°C treatment. This indicates that MC was less affected by 4°C treatment, and that it utilized its bark and woody tissue soluble sugars for buds, new leave, catkin growth, and development. Similar results were reported in peach after controlled freezing treatments; sugar content was lower after freezing temperature treatments (Yun et al., 2014). However, in our samples from an earlier natural freeze experiment, soluble sugar content was increased after low-temperature conditions (Kaur et al., 2023). This could be explained by the fact that excised branches in growth chambers are no longer attached to trees, limiting sugar translocation from lower plant parts to the more resource demanding shoot apex. In field conditions, higher bark soluble sugar content in apical portions after a freeze could be due to mobilization of sugar from lower plant parts to apical portions to support the higher sugar demand during low temperatures. This is consistent with the suggestion that carbohydrates can enhance cold hardiness or to support metabolic processes that confer cold tolerance (Morin et al., 2007). This is also consistent with the observation that apical bud growth is supported by the carbohydrate content near the shoot apex as well as non-structural carbohydrates transported by the vascular system (Tixier et al., 2017). Our research on pecan secondary buds, which can develop into pistillate and staminate flowers if the primary compound buds are killed, suggests that successful bud burst is promoted by carbohydrate levels stored in both the bark and wood (Zhang et al., 2022, Zhang et al, 2023).

Overall, KG, the most damaged combination compared to MC and PP, had higher soluble sugar content in both the bark and woody tissue. This could be due to differences in the growth stages of different scion/rootstock combinations. We collected branches from KG, MC, and PP on same day; during sampling time KG was a little behind in bud break, compared to more advanced growth on MC and PP, indicating these scion/rootstock combinations were using their sugars rapidly for new growth. Similarly, in controlled chamber conditions after low-temperature treatment, KG had the least growth in terms of bud break and growth, leaves, and catkin formation compared to other scion/rootstock combinations. Overall MC had lower soluble sugar content and had a better bud/leaf/catkin growth at all three growth stages. Similarly, when walnut was subjected to freezing conditions, a significant increase in sugar was observed in tolerant versus sensitive genotypes was observed (Panahi et al., 2021).

In stage I, the starch content in bark samples treated at -2°C, 0°C, 2°C, and 4°C for 8 hours was significantly higher than that of controls except 2°C in KG and PP (**Figure 7**). As the major carbohydrate reserve in trees starch may be utilized as a secondary source when demand exceeds the current photo-assimilate supply (Bustan et al., 2011). At stage II, in controls KG had significantly higher bark starch content than PP. At stage III in KG, PP, and MC, the starch content in bark samples treated at -2°C was significantly lower than controls, 0°C, and 4°C. At stage III, there were no significant variations among the scion/rootstock combinations in bark starch levels after all temperature treatment and controls. This could be a function of damage and slowed growth after being exposed to low temperatures at stage II and stage III compared to stage I. At the bud break stage, carbohydrates taken up by buds are derived from the mobilization of storage reserves (Alves et al., 2007; Simões et al., 2014). At this stage, glucose is generated from starch degradation or by the sucrose cleavage (Cho et al., 2018).

No significant variation was seen in woody tissue starch content among all the temperature treatments and scion/rootstock combinations at stage I and stage III (**Figure 9**). This is consistent with Wanjiku and Bohne (2016) report of no significant changes in the starch content of hazelnut cultivars after low temperature treatments. Similarly, in Eucalyptus no significant variation in starch content was observed in chamber (treated at 25, 16, 13, 10, 7, 4, and 0°C for a week) and field, conditions (Leborgne et al., 1995). However, we determined the starch content in woody tissue and bark from stage II, had significant variation among scion/rootstock combinations in the control treatment (**Figures 7**, **9**). This suggests differences in the growth rate of scion/rootstock combinations and utilization of starch among different scion/rootstock combinations.

## 5 Conclusions

Our results demonstrate that scion/rootstock combination, growth stage, and temperature affect susceptibility of pecan buds and shoots to spring freeze damage. Significant differences were detected in soluble sugar and starch levels in pecan bark and wood after low-temperature exposure between the treated and control branches. For example, the bark soluble sugar content was significantly lower in branches after the low-temperature treatments (-2°C for 8 hours) at stage I and stage II. The Maramec/Colby combination had minimum damage to leaves/bud/flowers and had lower bark soluble sugar and starch, and woody tissue soluble sugar, compared to the Kanza/Giles combination which had maximum damage and higher bark soluble sugar and starch, and woody tissue soluble sugar contents. These results indicate that Maramec/Colby was more tolerant to spring freeze damage than the other two pecan scion/rootstock combinations. It further suggests that pecan trees employ a strategy of reallocating stored sugar and starch reserves to achieve freeze tolerance. This research provides insights for scion/rootstock selection in orchard establishment decisions.

## Data availability statement

The original contributions presented in the study are included in the article/**Supplementary Material**. Further inquiries can be directed to the corresponding author.

## Author contributions

AK: Data curation, Formal analysis, Investigation, Writing – original draft, Writing – review & editing. LZ: Conceptualization, Project administration, Resources, Supervision, Writing – review & editing. NM: Conceptualization, Writing – review & editing. LF: Writing – review & editing. CG: Writing – review & editing. YS: Investigation, Writing – review & editing. SP: Investigation, Writing – review & editing. NP: Investigation, Writing – review & editing. MY: Writing – review & editing. JM: Resources, Writing – review & editing.
