# Supplementary material for: Dormant carbohydrate reserves enhance pecan tree spring freeze tolerance: controlled environment observations
Source: Front Plant Sci. 2024 May 22;15:1393305. doi: 10.3389/fpls.2024.1393305 (PMC11150881; doi:10.3389/fpls.2024.1393305)
Supplement: Supplementary file 1 [file Table_1.docx]

Supplementary Document

Table 1. Controlled temperature, humidity, and light conditions in the growth chambers.

| **Time** | **Temperature (F)** | **Temperature (℃)** | **Relative Humidity (%)** | **Light duration/intensity** |
| --- | --- | --- | --- | --- |
| 0:00 | 58.1 | 14.5 | 75.7 | 0 |
| 1:00 | 57.0 | 13.9 | 77.9 | 0 |
| 2:00 | 56.1 | 13.4 | 79.5 | 0 |
| 3:00 | 55.3 | 12.9 | 80.7 | 0 |
| 4:00 | 54.7 | 12.6 | 81.5 | 0 |
| 5:00 | 54.2 | 12.3 | 82.7 | 0 |
| 6:00 | 53.7 | 12.1 | 83.7 | 0 |
| 7:00 | 54.3 | 12.4 | 83.0 | 11 |
| 8:00 | 56.9 | 13.8 | 78.3 | 22 |
| 9:00 | 59.7 | 15.4 | 72.1 | 33 |
| 10:00 | 62.5 | 16.9 | 66.2 | 33 |
| 11:00 | 65.0 | 18.3 | 60.9 | 33 |
| 12:00 | 67.0 | 19.4 | 56.9 | 33 |
| 13:00 | 68.6 | 20.3 | 54.0 | 33 |
| 14:00 | 69.8 | 21.0 | 52.0 | 33 |
| 15:00 | 70.7 | 21.5 | 50.2 | 33 |
| 16:00 | 70.9 | 21.6 | 49.4 | 33 |
| 17:00 | 70.5 | 21.4 | 49.8 | 33 |
| 18:00 | 69.4 | 20.8 | 51.4 | 11 |
| 19:00 | 67.1 | 19.5 | 55.6 | 0 |
| 20:00 | 63.8 | 17.7 | 55.6 | 0 |
| 21:00 | 61.7 | 16.5 | 66.0 | 0 |
| 22:00 | 60.2 | 15.7 | 69.5 | 0 |
| 23:00 | 59.1 | 15.1 | 72.4 | 0 |
|  |  |  |  |  |
| **Light intensity: 0 - No light/lights off, 11 and 22- low light, 33-full light** | | | | |
